# Vascular Endothelial Growth Factor and Mesenchymal Stem Cells Revealed Similar Bone Formation to Allograft in a Sheep Model

**DOI:** 10.1155/2021/6676609

**Published:** 2021-03-03

**Authors:** Chris H. Dreyer, Niklas R. Jørgensen, Søren Overgaard, Ling Qin, Ming Ding

**Affiliations:** ^1^Orthopaedic Research Laboratory, Department of Orthopaedic Surgery and Traumatology, Odense University Hospital, Department of Clinical Research, University of Southern Denmark, Sdr. Boulevard 29, DK-5000 Odense, Denmark; ^2^Department of Orthopaedic Surgery and Traumatology, Slagelse Hospital, Region Zealand, Denmark; ^3^Department of Clinical Biochemistry, Rigshospitalet, Valdemar Hansens Vej 1-23, DK-2600 Glostrup, Denmark; ^4^Department of Clinical Medicine, Faculty of Health and Medical Sciences, University of Copenhagen, Copenhagen, Denmark; ^5^Department of Orthopaedics & Traumatology, The Chinese University of Hong Kong, Shatin, N.T., Hong Kong

## Abstract

**Introduction:**

Mesenchymal stem cells (MSCs) and vascular endothelial growth factor (VEGF) are key factors in bone regeneration. Further stimulation should establish an enhanced cell environment optimal for vessel evolvement and hereby being able to attract bone-forming cells. The aim of this study was to generate new bone by using MSCs and VEGF, being able to stimulate growth equal to allograft.

**Methods:**

Eight Texel/Gotland sheep had four titanium implants in a size of 10 × 12 mm inserted into bilateral distal femurs, containing a 2 mm gap. In the gap, autologous 3 × 10^6^ MSCs seeded on hydroxyapatite (HA) granules in combination with 10 ng, 100 ng, and 500 ng VEGF release/day were added. After 12 weeks, the implant-bone blocks were harvested, embedded, and sectioned for histomorphometric analysis. Bone formation and mechanical fixation were evaluated. Blood samples were collected for the determination of bone-related biomarkers and VEGF in serum at weeks 0, 1, 4, 8, and 12.

**Results:**

The combination of 3 × 10^6^ MSCs with 10 ng, 100 ng, and 500 ng VEGF release/day exhibited similar amount of bone formation within the gap as allograft (*P* > 0.05). Moreover, no difference in mechanical fixation was observed between the groups (*P* > 0.05). Serum biomarkers showed no significant difference compared to baseline (all *P* > 0.05).

**Conclusion:**

MSCs and VEGF exhibit significant bone regeneration, and their bone properties equal to allograft, with no systemic increase in osteogenic markers or VEGF with no visible side effects. This study indicates a possible new approach into solving the problem of insufficient allograft, in larger bone defects.

## 1. Introduction

In the case of trauma and reconstructive orthopaedic surgery, the need for a consistent method of regenerating bone is a priority, especially in large bone defects. An ideal biomaterial would bear three characteristics: osteoinductive, osteoconductive, and osteogenic properties [1]. Currently, allograft has been used for large surgical interventions and has been associated with mainly osteoconductive properties. Allograft has no osteogenic properties [2], with potential risk of disease transmission [3], and is a limited resource [4]. Therefore, finding an ideal biomaterial that can be produced in desired quantities and with no side effects would have a great impact on clinical outcomes, surgical complications, and economic perspectives [5].

To create the optimal environment for bone growth, the need for neutral surroundings made by vascularisation is essential. Blood supply is one of the most common limitations in the bone regeneration cycle [6]. Notably, the direct effect of vascular endothelial growth factor (VEGF) is to trigger angiogenesis and subsequent neovascularization. VEGF is reported to increase the proliferation and migration of endothelial cells by enhancing vessel permeability and tube formation during angiogenesis [7]. The indirect effect of the VEGF is the initiation of mesenchymal stem cells (MSCs) into following the osteogenic lineage [8, 9], which induces more osteoblasts at the healing site for a duel effect. While MSCs can be extracted from various tissues, MSCs derived from bone marrow (BMSC) have increased potency for bone regeneration with great potential in both animals and humans [10–12].

When seeded with MSCs, hydroxyapatite (HA) has previously been highlighted as a suitable carrier [13]. The rigidity and hardness of bone are due to the incorporation of mineral salt in the osteoid. Establishing the mineral complex with calcium and phosphate (i.e., HA), HA is ideal when compared to the human trabecular bone, which results in an osteoconductive scaffold. Even though it has been used for several decades, HA is still believed to be a promising substitute within the field of tissue engineering [14]. The VEGF was added to the titanium part of the implant, being able to stimulate the angiogenic stimulation around the implant. This is a well-known method when working with VEGF in bone formation, to add the stimulation of vessels on the titanium surface [12, 15].

The theory of optimising bone growth using a combination of MSCs and VEGF has previously been shown superior bone formation *in vivo* compared to using MSC alone [13, 16, 17]. The problem when using the combination treatment has been related to administration, release, and dosages [12]. A variety of methods for the local administration of VEGF have been investigated: from bioglass release [18], cement [19], or prolonging the release with hydrogel [20]. Up to date, the optimal method for the release of growth factors is limited and still being investigated.

The aim of this study was to investigate new bone regeneration by MSCs and VEGF compared to the gold standard of allograft. This was evaluated by combining different dosages of VEGF with MSCs on HA granules *in vivo* in a bilateral distal femur implant gap model in sheep for new bone formation. This study was based on *in vivo* and *in vitro* pilot studies, which were performed to provide an indication of the most suitable number of MSCs to use in this model combined with VEGF in this model.

We hypothesised that the effect of autologous MSCs can be stimulated by an additional VEGF coating to optimise the surrounding environment and increase the bone formation.

## 2. Materials and Methods

### 2.1. Animals

A total of 17 skeletally mature female sheep (ewes) of Texel/Gotland mixed breed were used: 8 for the *in vivo* pilot study, 1 for the *in vitro* pilot studies, and 8 for the current study. All sheep were of the same origin, and the observation was performed during the summer with observed temperature and the possibility to be inside a stable or outside in a field. The sheep received hay and water *ad libitum* as well as regular dietary chips for sheep in calculated dosages. The sheep were aged between 5 and 7 years and weighed 74 ± 22 kg (mean ± standard deviation (SD)). The acclimatisation period began one month prior to bone marrow aspiration (according to animal guidelines by the Biomedical Laboratory, University of Southern Denmark), and animals were housed at the Biomedical Laboratory 5 days after surgery procedures.

This experiment complies with the national, international, and institutional guidelines. The study was approved by the Danish Animal Experiments Inspectorate (no. 2012-15-2934-00704). Furthermore, the article complies with the ARRIVE guidelines.

### 2.2. Study Design

**Figure 1:**
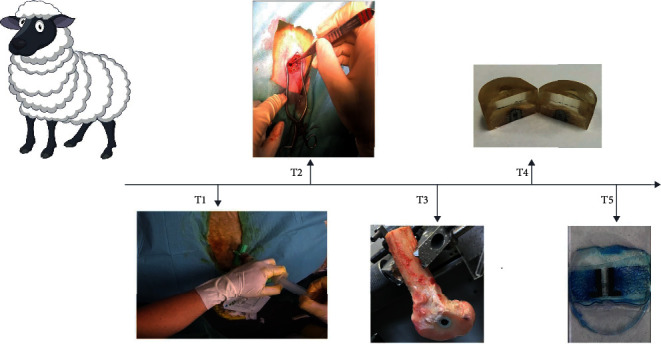
Illustration of the order of intervention. T1 (week 0): aspiration of bone marrow for the cultivation of MSC. T2 (week 4): surgical femoral implant gap model. T3 (week 16): preparation of samples for mechanical testing and embedding. T4 (week 28): embedded samples ready for sectioning. T5 (week 29): sectioned samples stained for quantification.

Ti-6Al-4V implant made of 90% titanium, 6% aluminium, and 4% vanadium at a size of 10 mm × 12 mm, with a 2 mm gap, was inserted in a critical size defect in the distal femur (Figure 1, T2+T3). The implants were placed on both the lateral and medial sides for a total of four implants in each sheep. Graft materials filled in the 2 mm gap of the implant and were isolated by a top washer to fixate the screw and material. The graft materials consisted of either the combination of MSCs and VEGF or allograft alone.

MSCs were seeded on the HA, and VEGF was coated on the sand-dusted neck surface of the titanium implant at the aforementioned doses. Blinded random allocation was only applied within each sheep. Thus, every sheep had four different locations containing three treatment groups and the control group—allograft. The total observation time for all *in vivo* groups in the pilot and current actual studies was 12 weeks.

### 2.3. Pilot Study

#### 2.3.1. In Vitro

This was performed to evaluate the influence of the coating materials and VEGF on the osteogenic capabilities of the MSCs. The study included three groups: Group A with only 0.5 × 10^4^ MSCs from normal skeletally mature ewes, Group B with only titanium implants containing a PDLLA-VEGF with 100 ng VEGF release/day, and Group C with the combination treatment of 0.5 × 10^4^ MSCs and PDLLA-VEGF coating with 100 ng VEGF release/day.

This study followed the same differentiation protocol of the MSCs as the *in vivo* portion. The cells were thawed and verified as confluent within 12 days. Group C was added to VEGF implants at day 12. Each group consisted of 4 analysis. The evaluation was performed by alizarin red staining after 18 days of seeding.

#### 2.3.2. In Vivo

This pilot study was performed to ascertain preferable MSC concentrations combined with VEGF for optimal new bone formation in 12 weeks. In total, eight skeletally mature ewes were used with the same surgical approach. The combination was 1 × 10^6^ cells, 3 × 10^6^ cells, and 5 × 10^6^ cells on 240 mg of HA combined with 10 ng VEGF release/day, respectively. The control in this pilot study was 3 × 10^6^ MSCs without any VEGF. Furthermore, one implant was empty (without MSC or VEGF) and one had only 10 ng VEGF release/day without HA or MSCs (Table 1). Due to an implant without any hydroxyapatite, the pilot study indicated the need to double the amount of HA within the gap.

### 2.4. Current Study

A total of eight ewes were used. The treated implants were randomly allocated within each sheep to receive 10 ng VEGF release/day, 100 ng VEGF release/day, and 500 ng VEGF release/day combined with bovine serum albumin (BSA) and 3 × 10^6^ MSCs seeded on HA (Table 2). Allograft served as the control treatment group and was consistent in each sheep as its own control. The total observation period was 12 weeks.

### 2.5. Vascular Endothelial Growth Factor

Out of several VEGF family members, rhVEGF165 was chosen for the study due to its elevated potency and effect [21]. This VEGF was added to the neck of the implant and was not in correlation with the seeded HA. VEGF was combined with BSA at a ratio of 1 : 50 due to BSA having a positive effect on the stabilisation of the protein (293-VE, R&D Systems), delivered in a firm structure of 500 *μ*g. It was released by a poly-DL-lactic acid (PDLLA) (R203, Sigma-Aldrich) coating with verified release within 3 weeks based on a Bradford curve and sterilised with gamma irradiation (Synergy Health Radeberg GmbH, STERIS, Germany) before surgery.

The release of VEGF/day can hereby be calculated into a total dosage of VEGF on each implant. The 10 ng VEGF/release/day is a total of 0.015 mg VEGF. The implants with a release of 100 ng VEGF/day had a total of 0.15 mg VEGF, and a release of 500 ng VEGF/release/day had a total of 0.525 mg VEGF.

### 2.6. MSCs from Bone Marrow

The autologous bone marrow was aspirated from the crista iliaca in four different locations for a total amount of 20 ml (Figure 1, T1). This was then transferred into four different 50 ml falcon tubes containing 4 ml of alpha minimum essential medium alpha (alphaMEMA, Life Technologies Europe BV, Denmark #22571-202) and 1 ml heparin. After the aspiration, the tubes were stored at +4°C.

The bone marrow was diluted with phosphate-buffered saline (PBS, Dulbeccos, Life Technologies Europe BV, Denmark #14190094), alpha MEM, fetal bovine serum (FBS, Sigma-Aldrich, Denmark #F0804), and penicillin-streptomycin glutamate (PSG, Life Technologies Europe BV, Denmark #10378016). The colony-forming unit (CFU) cultivation was stained with crystal violet blue and counted 14 days after aspiration.

The subculturing of the MSC began when the cells had 80–90% confluence. Cells were prepared with PBS, trypsin-ethylenediaminetetraacetic acid (EDTA, 0.05%, Life Technologies Europe BV, Denmark #25300-054), and MEM and then manually counted. The cells were then distributed in tubes of 5 × 10^5^ cells for the preparation to be seeded on the HA.

For storage of the cells, 10 ml syringes were cut open at the tip and placed in upright positions in the intubation chamber during the night before surgery. The syringes were then filled with 500 mg (±1 mg) hydroxyapatite and then added to a 100 *μ*l growth medium and cell suspension with 3 × 10^6^ cells [13].

The cells were verified as plastic-adherent after being differentiated in two full passages with a constant temperature of 37°C in an incubation locker, then stained with crystal violet blue for the verification of colony-forming units (CFU). No immunohistochemical analysis was performed. These were termed multipotent mesenchymal stem cells (MSCs) by the International Society for Cellular Therapy (ISCT) [22] and were referred to as MSCs in this paper.

### 2.7. Colony-Forming Unit

After the cells were diluted and isolated, 0.5–1 ml of cell suspension was prepared for CFU staining. A total of 1 × 10^5^ cells were then evaluated for CFU cultivation. The counting procedure followed previous guidelines using a BX47 microscope (Olympus, Tokyo, Japan) with a 4x lens [23].

### 2.8. Graft Material

The substitute used as a carrier for the MSCs consisted of pure HA granules. The diameter of each pore was between 1.0 and 2.5 mm (ENGIPORE, Fin-Ceramica, Faenza, Italy). This pore size would reach ~90% relative to the total volume and be transferred to the gap model in all sizes.

### 2.9. Surgical Procedure

All surgical procedures were performed at the Biomedical Laboratory at the University of Southern Denmark. Before handling, animals received 0.01 ml/kg of Rompun (xylazine hydrochloride, 20 mg/ml; Bayer Animal Health GmbH, Leverkusen, Germany). Bone marrow aspiration was performed under general anaesthesia with 3 mg/kg of propofol (10 mg/ml; B. Braun, Denmark), while surgical procedures were performed under general anaesthesia (2.0% isoflurane) using aseptic techniques including ethanol 70% and iodine vet (Kruuse, Denmark) for disinfection.

Bone marrow was aspirated, pooled, and inserted into the same sheep, herby performed autologously. The procedure was carried out under sterile conditions. Local analgesia of 5 ml (20 mg/ml) lidocaine was applied s.c. at all four aspiration sites. A bone marrow biopsy aspiration needle (13gax2-1/2in, Angiotech) penetrated the skin at the four sites located laterally from the spina iliaca posterior superior crest, and 3–4 ml of bone marrow was aspirated bilaterally (Figure 1, T1).

In the femoral gap model, the primary incision was placed at the lateral or medial condyle site, and the periosteal surface was exposed by an incision through the fascia with electrocauterisation splitting of the soft tissue. A low-speed drill created a 12 mm deep cylindrical hole with a circumference of 10 mm. To remove residual bone particles, the gap was rinsed with saline before insertion of the implants forming a gap of 2 mm. Subsequently, the concentric gap was randomly allocated to one of the four treatment groups, the gap was filled with substitute, and the top washer of the implant was fixated. Finally, the wound was sutured in three layers and wound plast was added (Kruuse, Denmark). Postoperative analgesia and antibiotics included 0.3 mg/ml buprenorphine (Temgesic, Denmark) and Curamox (150 mg/ml amoxicillin, Denmark), which was administered daily for 3 days. After 12 weeks, the sheep were euthanised with an overdose of Euthanimal (200 mg/ml, Alfasan, Netherland) and distal femurs were harvested bilaterally, stored at 20°, and thrawed for further processing based on an existing protocol [24].

### 2.10. Preparation

The bone-implant blocks with surrounding gap bone were divided into two parts using Exakt diamond band saw (Exakt Apparatebau) (Figure 1, T3): one 6.5 mm thick sample was dehydrated in graded ethanol (70–99%) at room temperature with electronic stirring and subsequently embedded in methyl methacrylate (Technovit 9100) (Figure 1, T4). After sectioning, the tissue was stained with toluidine blue O staining for histological analysis (Figure 1, T5). Another 3.5 mm thick sample was stored at -20° for mechanical push-out testing.

### 2.11. Histology

**Figure 2:**
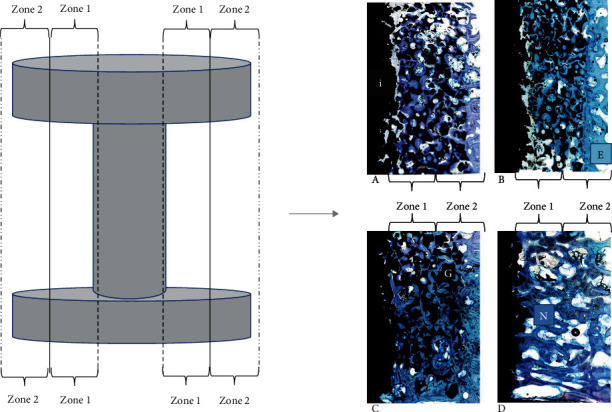
Illustration of the implant divided into zone 1 (close to the implant) and zone 2 (close to the existing bone). Histological images from the gap in each treatment and control group were stained with toluidine blue O after 12 weeks. A: MSC+VEGF 10 ng/day; B: MSC+VEGF 100 ng/day; C: MSC+VEGF 500 ng/day; D: allograft. These overview pictures were taken with newCAST (Visiopharm, Denmark; lens 4). E: existing bone; G: granula; i: implant; N: new bone.

The tissue within the region of interest (ROI) of the toluidine blue O-stained sections was classified as bone: blue coloured as osteocytes and fibrous tissue; purple with visible fibril fibres and low cell density as granula; black as the implant, miscellaneous, or marrow; and nonstained areas as adipose (Figure 2). Blinding of the treatment group and control treatment in the evaluation was difficult due to the histological characteristics of HA; however, treatment groups could not be distinguished and were blinded during evaluation.

### 2.12. Histomorphometry

Volume fractions of each tissue in the predefined ROI were measured by Cavalieri's principle using stereological software (newCAST, Visiopharm, Denmark). The ROI was the 2 mm gap (total volume, TV) of four sections from each implant and gave approximately 2000–3000 points pr. implant for representable results [25, 26]. Furthermore, the gap region was divided into two zones: close to the implant (zone 1) and close to the existing bone (zone 2).

### 2.13. Serum Biomarkers

Approximately 20 ml of blood was collected from the jugular vein at six different time points: at surgery day, 1 week, 2 weeks, 4 weeks, 8 weeks, and at euthanisation (12 weeks). Since the containment of MSCs and VEGF was the same within each sheep, results are compared to the preoperative baseline for any systemic effect. The blood was stored at 4°C for 30 minutes to clot before preparation. Then, it was centrifuged for 10 minutes with 4000 relative centrifugal force (RCF) at 4°C to produce 8–10 ml of serum. The serum was analysed for bone markers to detect the systemic activity of osteoblasts, osteoclasts, and VEGF to qualify any systemic effect from the stimulation during the early bone regenerative phase. Osteoprotegerin (OPG) Sheep OPG ELISA Kit (Cat. no.: MBS2506141), receptor activator and nuclear factor–jB ligand (RANKL) Sheep Receptor Activator of Nuclear Factor KB Ligand (RANKL) ELISA Kit, procollagen type-1 (PINP) Sheep Procollagen Type I N-Terminal Propeptide (PINP) ELISA Kit, Sclerostin, Sheep Sclerostin (SOST) ELISA Kit (Cat. no.: MBS033198), VEGF Sheep Vascular Endothelial Growth Factor (Cat. no.: MBS737944), Fructosamine Sheep Fructosamine ELISA Kit, Bioassay Technology Laboratory (Cat. no.: E0122Sh), and osteocalcin and carboxy-terminal collagen crosslink (CTX-I) by iSYS immunodiagnostic system IDS were used for measuring the bone biomarkers in serum.

### 2.14. Mechanical Testing

Following storage at -20°, the samples were placed at room temperature prior to mechanical testing for 2 hours to defreeze. The mechanical test was performed using the Mechanical Testing System (MTS, hydraulic material testing system; MTS Systems Co.). The 3.5 mm bone-implant block was placed on the specifically designed platen under a 6 mm diameter upper testing column. The preload was 3 newton, and the displacement rod was 5 mm/min. The compression force was applied to push the implant out of surrounding bone tissues. The force-displacement curve was recorded and converted to a stress-strain curve for calculating shear mechanical properties of the interphase between bone and implant. This provides measures of shear stiffness (MPa), failure energy (kJ/cm^2^), and shear strength (MPa) provided for bone breakthrough [27].

### 2.15. Statistical Analysis

One-way analysis of variance (ANOVA) was used to calculate overall differences between MSCs combined with 3 dosages of VEGF and compared to the control (allograft). Multiple comparisons were performed using the Holm-Bonferroni test (as appropriate) for normal distributions and the Kruskal-Wallis test for nonnormal distributions. *P* value less than 5% was considered significant. The graphs and statistics were measured and constructed in GraphPad Prism v. 7 (GraphPad Software, Inc.). The error of the first kind (*t*2*α*) was set at 1.96 with a confidence level of 95%. The critical value for the error of the second kind (*t*_*β*_) was 0.84 due to the selected power of 80%. The minimal relevant difference was selected at 70% and the SD at 50%. According to these assumptions, at least six implants should be included in each group. We included eight sheep (i.e., 8 implants) in each group to account for any illness or dropouts.

## 3. Results

### 3.1. Animals

The animals were observed daily by animal technicians. All sheep survived, and no sheep showed any signs of illness or stress during the experiment. The total of four implants did not contain any HA in the 2 mm gap on the histological section, due to the fitting and structure of the granule blocking each other for an even distribution. This caused a lack of seeded MSCs within the gap, and these implants were excluded. No group had less than six samples according to the power calculation.

### 3.2. Colony-Forming Unit

The CFU from all donor sheep was 51.3 colonies per 1 × 10^5^ cells (SD ± 15.1). The bone marrow was aspirated and implanted autologously.

### 3.3. Histology

Staining with toluidine blue O showed generally new bone formation within the entire gap surrounding the implant. Furthermore, there was ingrowth to both the implant and existing bone for promising osteointegration (Figure 2). Polarised light determined the placement of the collagen lamellae to verify mature bone with regular alignment.

### 3.4. Pilot Studies

#### 3.4.1. In Vitro Results

The staining in the *in vitro* study indicated positive reactions in both the MSC alone and in the combination treatment with VEGF, though no obvious differences were observed between the groups. VEGF alone had a very limited reaction to the staining.

#### 3.4.2. In Vivo Results

The *in vivo* pilot study indicated the same bone formation in the 3 × 10^6^ and 5 × 10^6^ MSC groups in combination with VEGF, and less bone formation when using MSCs alone. Moreover, the empty implant showed no bone formation and characterised the size of the defect as a critical size defect (CSD).

### 3.5. Histomorphometry

**Figure 3:**
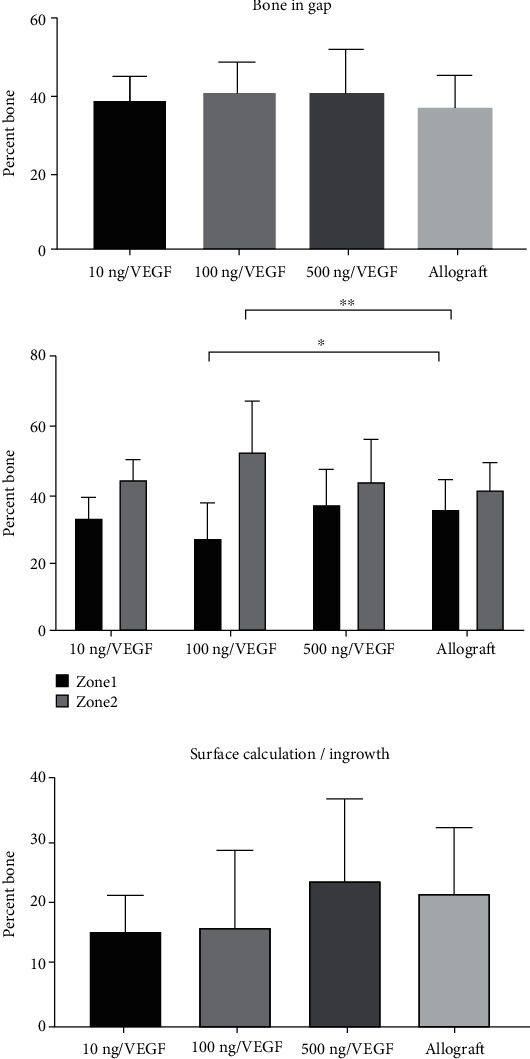
Percentage of bone formation within the gap based on histological images. No statistical differences were observed between the groups in overall bone within the gap or in the bone ingrowth. 100 ng/VEGF had a lower percentage in zone 1 (^∗^*P* > 0.05) and a higher percentage in zone 2 compared to allograft (^∗∗^*P* < 0.001).

The bone volume within the gap (Figure 2) showed no significant difference between the intervention groups and the allograft (*P* > 0.05) (Figure 3).

The group using 0.15 mg VEGF had significantly lower bone formation in zone 1 (*P* < 0.05), but significantly higher bone formation in zone 2 (*P* < 0.001) than the allograft group. The bone ingrowth showed no difference to the control (*P* > 0.05) (Figure 3).

### 3.6. Serum

**Figure 4:**
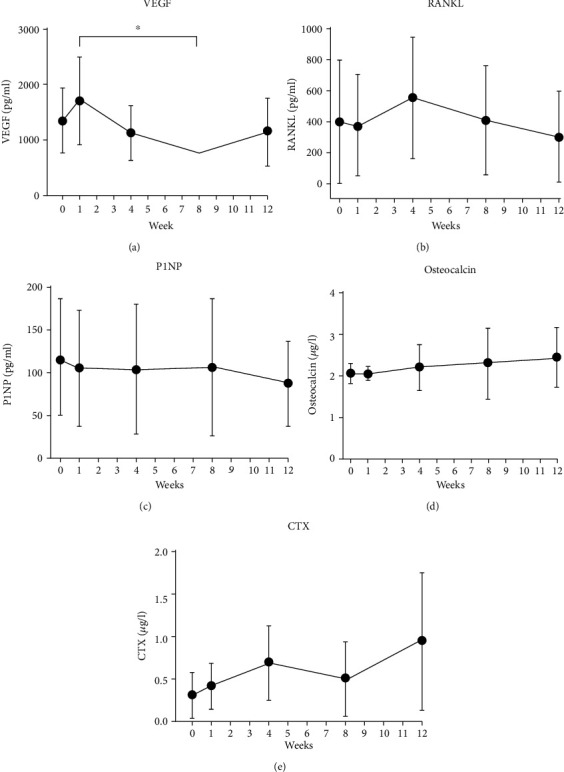
Blood serum samples at different time points: (a) vascular endothelial growth factor (VEGF); (b) receptor activator and nuclear factor–jB ligand (RANKL); (c) procollagen type-1 (P1NP); (d) osteocalcin; (e) carboxy-terminal collagen crosslink (CTX). ^∗^*P* > 0.05.

The bone markers that compared the preoperative baseline to 4 time points revealed no significant differences between before surgery and doing the regeneration phase, until euthanization (Figure 4).

However, a tendency of decreasing VEGF and RANKL was observed after week 1 with increasing osteocalcin and CTX. Moreover, VEGF exhibited a significant decrease from week 1 to week 8. Sclerostin could not be measured by the provided kit and was thus excluded from the analysis. OPG values were all below the minimum for the kit (i.e., <0.78 pg/ml).

### 3.7. Mechanical Testing

**Figure 5:**
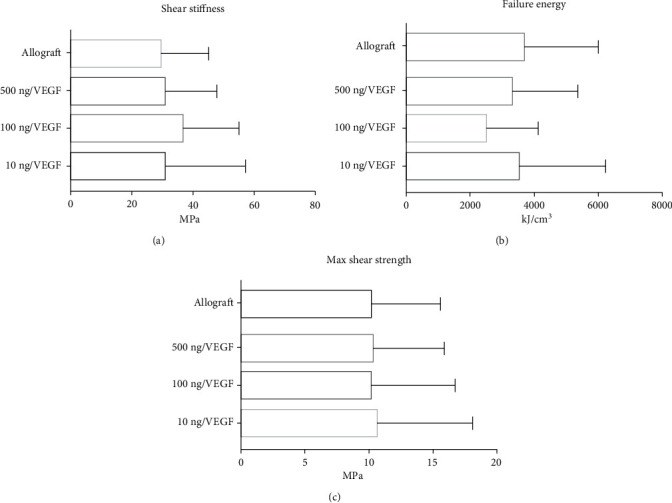
Mechanical properties from all groups defined by three parameters: shear stiffness, failure energy, and shear strength; MPa (megapascal), kJ/m^2^ (kilojoule/square centimetre). No statistical differences were observed between each group in any parameters measured.

When trying to apply pressure to the implants to verify the strength of the bone ingrowth, there were not any significant differences between using MSC and VEGF in different doses and allograft. The compared parameters were shear stiffness, failure energy, and shear strength (*P* > 0.05) (Figure 5).

## 4. Discussion

The aim of this study was to investigate new bone generation by MSCs and VEGF compared to the gold standard of allograft. This 12-week study revealed new bone generation by the combination of 3 × 10^6^ MSCs with 0.015 mg VEGF, 0.15 mg VEGF, or 0.525 mg VEGF and compared it to the golden standard of allograft. No statistical differences were observed when combining 3 × 10^6^ MSCs with 0.015 mg VEGF, 0.15 mg VEGF, or 0.525 mg VEGF, comparing to using allograft alone, neither in histological sections nor mechanical testing. Moreover, serum samples showed no systemic effect of the stimulation.

### 4.1. The Combination of MSC and VEGF

The use of combining MSCs and VEGF has been reported for bone formation in the past, and the research community continues to seek the optimal strategy for optimal bone formation in critical size defects [16, 28]. While factors such as BMP-2, BMP-7, IL-1, IL-6, TGF, and FGF at different dosages and combinations have been attempted using different methodologies, they have exhibited a weak impact on clinical procedures. Furthermore, the use of various stem cell types (e.g., bone marrow stem cells (BMSC), adipose-derived stem cells (ADSC), muscle-derived stem cells (MDSC), and embryonic stem cells (ESC)), different types of scaffolds (e.g., 3D printing, titanium, and magnesium), and indirect stimulation with mRNA, calcitonin gen-related protein (CGRP), or signalling pathways (e.g., Wnt) has also been tried. The possibilities related to the absolute effect on bone defects are endless [29, 30], receiving different results. Using MSC and VEGF has shown a good effect, for example, in a femur defect in a rat with a postoperative injection of VEGF from Gao et al. [16] and in a calvarial rat model from Subbiah et al. [31] but also in larger animals like the dog mandible defect from Khojasteh et al. [32].

These results are dependent on the animal model, defect, release method, and dosages. This is why this study is based from previous studies in a smaller animal model, hopefully being able to make a method available for translation between locations and species.

### 4.2. Design Based on Previous Publications

We previously performed the combination of BMCs and VEGF and the methods of cultivating and verifying the plastic-adherence capabilities of MSCs in a mouse model [13]. In this study, we attempted to translate this design into a large animal model. Our results showed the same bone-forming quality and quantitative effect as allograft, hereby verifying the potential of combining of BMCs and VEGF for further exploration towards translational practice. Of note, the difficult part of working with VEGF is the release, which is due to its short *in vivo* half-life time. This was combined with the low reproducibility of results, which might affect the total dosage and release period in existing designs [12, 33]. This negates the need for a suitable carrier and reasonable dosage when used for protein to have an optimal effect on bone formation.

The dosages of VEGF used were inspired by our previous design in severe combined immunodeficiency (SCID) mice, and the same dosage of 0.015 mg VEGF combined with BSA [13] was used in this design. The other dosages were ×10 and ×25 of this initial dosage due to the translation into a defect in a larger animal model. Surprisingly, there was no statistical difference between the treatment groups of different VEGF dosages. This could be due to the combination treatment with stem cells that the VEGF therapeutic window in this type of design will widen in reaction to additional cell activity.

### 4.3. Translational Potential

The methodology in these designs is very dependent on being simple, feasible, and cheap in order to increase the chance of implementation [34]. Working with differentiated stem cells requires a greater focus on elective procedures rather than acute surgery. This gap design does not indicate any specific clinical issue but provides a proof of concept effect of the trabecular bone structure, which is well represented in the spine, for example. Furthermore, due to the strong nature of HA, this design can provide instant stability [35]. The titanium implant has a porous plasma-sprayed structure that is similar to the general stems and cups used in arthroplasties. This makes the design feasible for testing bone ingrowth and stress shielding for translational purposes.

The crosstalk of osteoblast (OB) cells differentiated from MSC and angiogenesis is of great impact to new bone. Interestingly, MSC and endothelial cell (EC) interaction studies have shown increased bone regeneration [36]. Some research groups have concluded that the combination treatment of MSC and VEGF is necessary to achieve the optimal bone-forming effect of VEGF [37, 38], whereas other results indicate that VEGF can be used both solely and in combination with MSC, with the focus on bone formation [39].

Despite this potential, in its evaluation of the last 10 years, no existing techniques have reached clinical trials to date.

### 4.4. Considerations about Allograft as Control

The control and mentioning of allograft as the gold standard are an ongoing discussion in tissue engineering in case of osteogenic properties [4, 40]. We based our choice by recent published results, where no significant difference was concluded when using autograft and allograft in bone remodelling in a small animal model [41]. Furthermore, allograft does not cause any further invasive procedures or possible side effect in both the human and the animals as needed when harvesting autograft bone.

### 4.5. The Strengths

This study was related to its reproducible design. Our research group has made the femoral gap model several times and thus enhanced the quality of operation and evaluation each time [24, 27]. Moreover, cell aspiration and cultivation followed strict and reproducible protocols, and dosages with combination treatment have been tested in a previously published article. The handling of the MSCs in all our studies was performed using the same experienced laboratory technician. The blood sampling did not focus on specific dosages for a systemic effect; instead, it explained the systemic reaction in the development of bone growth and release of all of MSC and VEGF to verify possible uses for further translational studies.

### 4.6. The Limitations

This study included the low amount of test subjects and implants, which reduced the power of the results. However, this study meets the power calculation and further considers the 3 Rs in the model by using several implants per sheep. Furthermore, the amount of HA required to fill in the gaps was increased from our pilot study to the current study. Unfortunately, some sections did not contain any HA with MSCs and were thus excluded from Results. The MSC only treatment group from the *in vivo* pilot study could not be evaluated by statistics, as the HA amount had such a small area in the gap, hereby not distributing the cells sufficiently. These results could be due to variation in granule size as opposed to the total weight of the substitute, which should be considered when fitting different granule sizes into a predefined gap.

## 5. Conclusion

The study showed that the combination of MSCs and VEGF had the same bone healing and mechanical fixation strength as implants treated with allograft. Moreover, systemic serum biomarkers showed no change in bone markers compared to baseline at any studied time point and level of VEGF stimulation.

Based on these results, we suggest that future studies consider the combined effect of BMCs with VEGF to stimulate the angiogenic environment in bone growth. This could, with adaption into a specific defect, be a strategy to solve the issue of a larger bone defect, even combining with new technology such as 3D printing.

## Figures and Tables

**Table 1:** Grouping of *in vivo* pilot study.

	HA	MSC	VEGF
Group 1—7 implants	240 mg	3 × 10^6^	—
Group 2—8 implants	240 mg	1 × 10^6^	10 ng VEGF release/day
Group 3—8 implants	240 mg	3 × 10^6^	10 ng VEGF release/day
Group 4—8 implants	240 mg	5 × 10^6^	10 ng VEGF release/day
Group 5—1 implant	—	—	—

**Table 2:** Grouping of *in vivo* primary study.

	HA	MSC	VEGF
Group 1—8 implants	500 mg	3 × 10^6^	10 ng release/day
Group 2—8 implants	500 mg	3 × 10^6^	100 ng release/day
Group 3—8 implants	500 mg	3 × 10^6^	500 ng release/day
Group 4—8 implants	500 mg	Allograft	—

## Data Availability

The data for this study were analyzed by histomorphometry, and all data are stored at the Orthopaedic Research Laboratory, Department of Orthopaedic Surgery and Traumatology, Odense University Hospital, Department of Clinical Research, University of Southern Denmark in datafiles from Visiopharm, Denmark, verifying every count and statistics made for the analysis included in this study. Blood serum samples were gathered at the Department of Clinical Biochemistry, Rigshospitalet, with stored information.
